# Biological and Metabolomic Characterization of Human Dermal Fibroblasts and Mesenchymal Stem Cells Derived from Human Dental Pulp and Adipose Tissue: a Pilot Comparative Study

**DOI:** 10.1007/s12015-025-10970-0

**Published:** 2025-09-10

**Authors:** Zuzana Hatoková, Bibiána Baďurová, Martin Kertys, Nela Žideková, Andrea Evinová, Lucia Kotúľová, Marián Grendár, Denisa Harvanová, Lucia Slovinská, Erika Halašová, Henrieta Škovierová, Slavomíra Nováková

**Affiliations:** 1 Biomedical Centre Martin, Jessenius Faculty of Medicine in Martin, Comenius University in Bratislava, Malá Hora 4C, Martin, 036 01 Slovakia; 2 Department of Medical Biochemistry, Jessenius Faculty of Medicine in Martin, Comenius University in Bratislava, Malá Hora 4D, Martin, 036 01 Slovakia; 3 Department of Pharmacology, Jessenius Faculty of Medicine in Martin, Comenius University in Bratislava, Malá Hora 4C, Martin, 036 01 Slovakia; 4 Associated Tissue Bank, Faculty of Medicine, Pavol Jozef Safarik University and Louis Pasteur University Hospital, Trieda SNP 1, Košice, 040 11 Slovakia

**Keywords:** Human adult mesenchymal stem cells, Human dermal fibroblast adult, Targeted metabolomics, Differentiation, High-resolution respirometry, Cell-based therapy

## Abstract

**Background:**

Several studies have suggested that adult human dermal fibroblasts (HDFa) may be a potential alternative source to mesenchymal stem cells for cell therapies. This study aims to characterize HDFa, adipose-derived stem cells (ADMSCs) and dental pulp stem cells (DPSCs) to investigate their proliferation, differentiation potential, mitochondrial respiration, and metabolomic profile. We identified molecules and characteristics that would differentiate MSCs from different sources or confirm their uniformity.

**Methods:**

Differentiation was induced using osteogenic and adipogenic differentiation media. Proteins specific to each differentiation process were monitored by immunofluorescence staining. High-resolution respirometry and targeted metabolomic analysis using the AbsoluteIDQ^®^p180 kit (Biocrates Life Science) were applied to identify the essential properties of the studied cells.

**Results:**

HDFa cells, ADMSCs, and DPSCs demonstrated morphological characteristics of mesenchymal stem cells (MSCs). In general, DPSCs and HDFa showed significantly higher proliferation than ADMSCs. Osteogenic and adipogenic capacities were similar for all cell origins after 21 days, but ADMSCs exhibited earlier calcium deposit formation. FoxO1 and adiponectin as osteogenic and adipogenic-related proteins confirmed differentiation processes. High-resolution respirometry and metabolomic analysis showed potential distinguished characterization, mainly for DPSC cells. Our results also demonstrated that lipid profiling could be a promising tool for MSC characterization.

**Conclusions:**

Our analyzed data suggest that HDFa have properties similar to DPSCs and ADMSCs. However, each cell type has been shown to have unique specific characteristics. The similarities and differences in the characteristics of HDFa, ADMSCs, and DPSCs should be studied in detail and in a larger cohort when planning stem cell-based therapy.

**Graphical Abstract:**

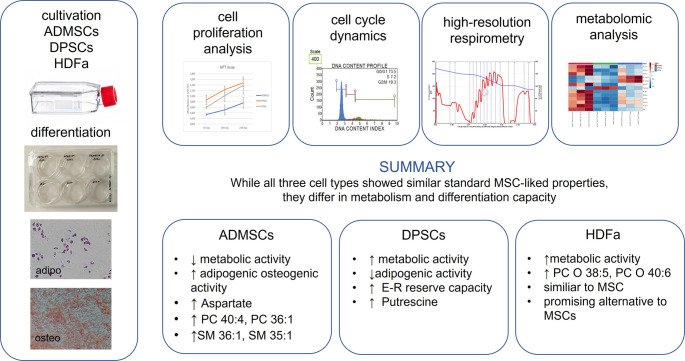

## Introduction

Recently, stem cell research has gained significant attention due to the promising potential of stem cells in regenerative medicine and tissue engineering [1]. Mesenchymal stem cells (MSCs) are multipotent cells recognized for their ability to self-renew and differentiate into various cell types [2], as well as for their role in immune regulation [3]. The MSCs were initially isolated from the bone marrow, but they have also been identified from various tissues, including adipose tissue (ADMSCs) and dental pulp (DPSCs) [4]. Dental stem cells can be derived from oral tissues, including dental pulp, periodontal ligaments, and exfoliated teeth [5]. Among them, dental pulp stem cells (DPSCs) stand out due to their high proliferation rate, multilineage differentiation potential, including the ability to differentiate into osteoblasts, adipocytes, chondrocytes, and neurons, and their demonstrated effectiveness in craniofacial and neural tissue regeneration [6]. Recent studies further support their use in dental tissue engineering, demonstrating that their odontogenic differentiation can be enhanced by using extracellular vesicles and epigenetic modulators such as 5-Aza-2′-deoxycytidine, particularly when combined with decellularized scaffolds, highlighting their strong potential for endodontic regeneration [7]. Conversely, stem cells obtained from adipose tissue have attracted significant interest because they are plentiful and can be easily collected through non-invasive techniques. ADMSCs also exhibit a strong capacity for proliferation and differentiation into multiple lineages, including osteogenic, chondrogenic, and adipogenic, making them highly promising for soft tissue repair and cosmetic surgery [8]. Additionally, human dermal fibroblasts (HDFa), while not classified as stem cells, play a key role in maintaining skin integrity, producing extracellular matrix components such as collagen, and facilitating wound healing. HDFa cells are morphologically similar to MSCs, and their properties meet the characteristics of MSCs according to ISCT (International Society for Cell and Gene Therapy) [9]. They represent a promising alternative to mesenchymal stem cells due to their accessibility, ease of isolation, and reprogramming potential into pluripotent or lineage-specific cell types [10]. Their scalability and ethical advantages position them as a valuable resource for advancing stem cell research, tissue engineering, and regenerative medicine, mainly where traditional mesenchymal stem cell sources are limited or difficult to access [11, 12]. While the fundamental characteristics of these cells are well recognized, a detailed comparison of their biological properties across several key parameters is necessary to optimize their application in clinical settings. A comprehensive evaluation of ADMSCs, DPSCs, and HDFa across several critical biological processes: differentiation capacity, proliferation rates, cell cycle regulation, respiration (mitochondrial activity), and metabolomics profiles can provide crucial information for their therapeutic applications. Their differentiation potential shows the ability of stem cells to heal injured tissues. At the same time, cell growth rates indicate how well these cells can multiply and replace specific tissues. Understanding cell cycle dynamics offers valuable information about the efficiency of cell duplication, which is vital for regenerative uses that demand quick cell replacement [13]. The identification of changes in cell cycle patterns, precisely the duration of individual phases like G0/G1, S, and G2/M, may affect not only the rates of cell division but also their ability to differentiate and survive over time in culture [14]. Beyond these cellular characteristics, respiration and metabolomic profiles are also recognized as essential for the optimal functioning of stem cells. Mitochondrial respiration reflects the energy metabolism of cells, which is critical for supporting the high energy demands of cell proliferation and differentiation. Differences in energy metabolism, particularly oxidative phosphorylation (OXPHOS) and glycolysis can influence stem cell differentiation and proliferation capacities [15–17]. Metabolomic analysis of cells has become an important technique for studying cellular biochemistry [18]. DPSCs, ADMSCs, and HDFa may rely on different metabolic pathways, which could have significant implications for their application in regenerative medicine, especially in their ability to sustain long-term functionality or adapt to different tissue environments, particularly in environments with metabolic stress or tissue damage. Metabolic profiling is a valuable tool for analyzing small molecules within biological samples, providing insights into how various cell types respond to environmental stimuli. This approach is particularly crucial for the optimization of cell-based therapies. This study provides a clearer understanding of the advantages and limitations of individual cell types, which will facilitate their use in regenerative medicine and tissue engineering.

## Materials and methods

### Cultivation of Cells

ADMSCs used in this study were isolated from the subcutaneous adipose tissue of three donors, and verified using the method described by Spakova et al. [19]. Briefly, adipose tissue was washed in PBS (Sigma Aldrich), cut into small pieces and digested with 1.0 mg/mL collagenase type I (Gibco) in low glucose DMEM containing 1% (v/v) antibiotic/antimycotic solution overnight at 37 °C under continuous rolling. The digested fragments were then filtered through a 70 μm cell strainer (BD Falcon™, Biosciences) and cultured in vitro. The informed consent was observed from all the donors involved in the study (Approval ID 2020/EK/09067). Cryopreserved cells of DPSCs (Lonza) and HDFa (Gibco) from three different donors were purchased. Cells were cultured in the same conditions as the ADMSC cells. All cell lines were maintained in DMEM: F12 + GlutaMAX medium (Gibco) supplemented with 10% fetal bovine serum (FBS, Biosera) and 1% penicilin-streptomycin (Biosera) under a 5% CO_2_ humidified atmosphere at 37 °C. The medium was changed every 3 days until the cells reached approximately 80% confluence. Cells were harvested by incubating them with TrypLE Express Enzyme (Gibco) for 5 min at 37 °C, followed by washing and centrifugation at 400 x g (5 min, 13 °C). Cells between 4th and 5th passages were used in the experiments. Cell morphology was monitored using light microscopy (Optica). Flow cytometry confirmed that all cell sources expressed key mesenchymal stem cell surface markers CD73, CD90, and CD105, while lacking expression of the hematopoietic marker CD45 [20].

### MTT Assay

Cells were seeded in 96-well plates (Corning) in hexaplets at 5 × 10^3^ cells/cm^2^ density. The proliferation of cells was monitored on the following days: 7, 14, and 21. At each time point, the cells were washed with Dulbecco’s PBS solution (DPBS, Gibco) and incubated with mixture of MTT (3-(4,5-dimethylthiazol-2-yl)−2,5-diphenyltetrazolium bromide solution, Duchefa Biochemie) and culture medium in ratio 1:10 for 5 h at 37 °C in a incubator with humidified atmosphere (5% CO_2_, 21% O_2_). Following incubation, 10% sodium dodecyl sulfate (SDS, Sigma) was added to each well. Then, plates were incubated overnight under the controlled conditions reported above. The absorbance was measured at 570 nm using a microplate reader (BioTek Technologies). All tests were performed in triplicate.

### Adipogenic and Osteogenic Differentiation

Cells were seeded in 6-well plates (Greiner) at 5 × 10^3^ cells/cm^2^ density. When cells reached 60% confluence, they were washed with DPBS (Gibco). StemPro^®^ Adipogenesis Differentiation Medium (Gibco) was applied for adipogenic differentiation. OsteoMAX-XF™ Differentiation Medium (Merck) was used for osteogenic differentiation. Differentiation media were prepared according to the manufacturer’s instructions. Cells cultured in standard growth medium were used as the control group. Differentiation and control media were changed every 3–4 days during incubation (21 days). The cells were maintained and differentiated into adipocytes and osteocytes under controlled conditions (5% CO_2_, humidified atmosphere at 37 °C). Morphological changes were assessed at 7, 14, and 21 days post-treatment.

Lipid droplet accumulation was detected using Oil Red O staining (Sigma-Aldrich) according to the manufacturer’s protocol with slight modifications. Briefly, cells were washed twice with DPBS (Gibco) and fixed with 4% paraformaldehyde (PFA, Cell Signaling Technology) for 30 min at room temperature in the dark. After fixation, cells were washed twice with distilled water and then incubated in 60% isopropyl alcohol (Sigma) for 5 min. They were stained with 0.3% Oil Red O solution (Sigma) for 15 min at room temperature on an orbital shaker in the dark. The 0.3% Oil Red O working solution was freshly prepared and filtered for each experiment. Following staining, cells were washed five times with distilled water. Lipid droplets were visualized under a light microscope (Optica). Quantification of stained Lipid droplets was performed after eluting the dye. After washing the cells once with 60% isopropyl alcohol, 100% isopropyl alcohol was added to the plates, and the cells were incubated for 5 min at room temperature on an orbital shaker in the dark. Absorbance was measured in duplicate at 510 nm using a microplate reader (BioTek Technologies). Absorbance was normalized to the total amount of proteins isolated from fixed cells.

Alizarin Red S staining was used to detect calcium deposits produced by osteocytes. Cells were washed twice with DPBS (Gibco) and fixed with 4% PFA (Cell Signaling Technology) for 30 min at room temperature in the dark. After fixation, cells were washed twice with DPBS and incubated in Alizarin Red (Merck) for 3 min at room temperature in the dark. After removing the dye, the cells were washed twice with distilled water. The mineralized extracellular matrix was visualized under a phase-contrast microscope (Optica). Afterwards, cells were incubated with a destaining solution (10% acetic acid, 20% methanol) for 15 min. Absorbance was measured in duplicate at 405 nm using a microplate reader (BioTek Technologies). Absorbance was normalized to the total amount of proteins isolated from fixed cells.

### Protein Isolation from Fixed Cells

The proteins were isolated following the procedure described by Rodrígez-Rigueiro et al. with modifications [21]. Briefly, the fixed and destained cells were washed twice with cold DPBS. The lysis buffer consisted of 4% SDS (Sigma), 0.1 M Tris-HCl, 100 mM dithiothreitol (DTT, Thermo Scientific), and protease inhibitors (Roche). The lysates were sonicated two times for 10 s. Homogenates were initially incubated at 100 °C for 30 min, followed by incubation at 80 °C for 2 h on a shaker, and then centrifuged (12 000 x g) at room temperature. The supernatants were collected and stored at −80 °C until use. Protein concentrations were determined by Pierce™ 660 nm Protein Assay (Thermo Scientific), according to the manufacturer’s instructions.

### Immunocytochemistry

Twenty-one days after applying adipogenic, osteogenic, and control media, the cells were washed with DPBS and fixed in 4% PFA for 30 min in the dark at room temperature. Then, the cells were incubated in a blocking buffer (5% goat serum (Canvax) in DPBS with 0.2% Triton™ X-100 (Sigma)) for 60 min at room temperature. Immunostaining was performed overnight at 4 °C and using followed antibodies: anti-ADIPOQ (Sigma-Aldrich, #HPA051767, dilution 1:50), FoxO1 (Cell Signaling Technology, #14952, dilution 1:50), anti-OPN (Sigma-Aldrich, #HPA027541, dilution 1:50). The cells were washed three times with washing buffer (DPBS with 0.2% Triton™ X-100) to remove unbound primary antibodies. Goat anti-rabbit Alexa Fluor™ Plus 488 (Abcam, # Ab 150077, dilution 1:500) and goat anti-mouse Alexa Fluor™ Plus 488 (Abcam, #Ab150113, dilution 1:500) were used as secondary antibodies. The nuclei were stained with 4’,6-diamidino-2-phenylindole (DAPI, Sigma). Fluorescence microscopy was performed using a WiScan^®^ Hermes instrument (IDEA Bio-Medical).

### Cell Cycle Analysis

Cell cycle analyses were performed using a Muse™ Cell Cycle Assay Kit in combination with the Muse™ Cell Analyzer (Luminex Corporation). The study was conducted according to the manufacturer’s protocol. When the cells reached approximately 80% confluence, they were detached using TrypLE Express Enzyme (Gibco) for 5 min at 37 °C. Detaching was followed by washing and centrifugation at 300 x g (5 min, 13 °C). The resulting cell pellets were rinsed with DPBS and fixed in cold ethanol. Fixed cells were stored at −20 °C until further analysis. On the day the analysis was performed, fixed cells were treated with Muse Cell Cycle reagent for 30 min in the dark. The proliferation index (PI) was calculated using the following formula [22]: $\:PI\:\left(\%\right)=\frac{(S+G2/M)}{(G0/G1+S+G2/M)}\:\times\:100\%.$

### High-Resolution Respirometry

Mitochondrial respiration in the cells was assessed using high-resolution respirometry (HRR) in a two-chamber system O2k-FluoRespirometer (Oroboros Instruments) on the day the cells reached approximately 80% confluence as described by Evinova et al. [23]. The cells were harvested as reported above. Measurements were performed in mitochondrial respiration medium MiR05-Kit (Oroboros Instruments) Maintained at 37 °C and stirred at 750 rpm. The coupling control protocol (CCP) was applied [24]. Basal respiration (ROUTINE, R), representing the physiological respiratory activity of cells, was recorded after titrating the cells into the chambers containing the mitochondrial respiratory medium. Pyruvate (final concentration 10 mM) was added as a substrate for cellular respiration, and oligomycin (2.5 µM), as an ATP synthase inhibitor, was used to induce a LEAK state (L). Afterwards, the maximal electron transport (E) capacity of the respiratory system was determined by stepwise titration with the uncoupler carbonyl cyanide 3-chlorophenylhydrazone (CCCP) in a concentration of 0.5 µM. To further analyze respiratory chain components, rotenone (0.5 nM) was added as a Complex I inhibitor, and Succinate (10 mM), a specific substrate for Complex II, was titrated. Succinate-stimulated respiration was observed in permeabilized cells following the addition of digitonin (0.8 µL per 6 million cells). Cytochrome c was titrated to assess the integrity of the outer mitochondrial membrane. Finally, Antimycin A (2.5 mM), a complex III inhibitor, was added to measure residual oxygen consumption (Rox) and nonmitochondrial respiration [25]. Measurements were normalized to the total amount of cells. Traces were analyzed using DatLab 7 software (Oroboros Instrument). Results were evaluated against residual oxygen consumption (Rox normalized). Each measurement was repeated three times.

### Metabolomics

#### Cell Lysate Preparation

Metabolomic analysis was performed using AbsoluteIDQ^®^p180 kit (Biocrates Life Science). Cell samples were prepared according to the manufacturer’s instructions. Briefly, when the density of the cells reached approximately 80%, the cells were washed with DPBS solution. Subsequently, the cells were trypsinized using TrypLE Express Enzyme (Gibco) and centrifuged at 452 x g for 3 min at 4 °C. The resulting cell pellets were frozen at −80 °C until lysis. Frozen pellets were thawed on ice and resuspended in 100% isopropanol [26]. Then, samples were sonicated in an ice bath for 3 min and frozen in Liquid nitrogen for 30 s. This freeze-thaw cycle was repeated three times. The samples were centrifuged at 31 500 x g for 5 min at 2 °C. The supernatants were transferred to vials and stored at −80 °C until further measurement and analysis.

#### Measurement and Data Analysis

A targeted metabolomics approach using the AbsoluteIDQ^®^ p180 kit (Biocrates Life Science) was selected to quantify metabolites. This approach allowed the simultaneous quantification of 185 analytes from six biochemical classes. Liquid chromatography-tandem mass spectrometry (LC-MS/MS) was used for the quantification of small molecule metabolites (amino acids and biogenic amines) and flow injection-tandem mass spectrometry (FIA-MS/MS) for the quantification of lipid-related molecules (acylcarnitines, phosphatidylcholines, etc.). The analytical system consisted of the Acquity UPLC™ I-Class liquid chromatography (Waters), composed of the flow-through needle sample manager, the binary solvent manager pump and the column manager coupled with the XEVO TQ-S triple quadrupole mass spectrometer (Waters). Separation of metabolites from LC-MS/MS analysis was done on ACQUITY UPLC™ BEH C18 (2.1 mm × 75 mm; 1.7 μm) column fitted with an ACQUITY UPLC™ BEH C18 VanGuard pre-column (column and pre-column were purchased from Waters). The MS/MS signals were integrated using MassLynx software version 4.2 (Waters). The data from MassLynx was subsequently analysed using WebIDQ software (Biocrates Life Sciences), an integral part of the kit.

The kit was prepared according to the manufacturer’s protocol. Briefly, 20 µL of lysate samples were pipetted on the filter spot of a 96-well extraction plate and dried under a gentle stream of nitrogen. During the next step, amino acids and biogenic amines were derivatised with 5% phenyl isothiocyanate (Sigma-Aldrich) and dried again. Metabolites were extracted with 5 mM ammonium acetate in methanol (Sigma-Aldrich), and the extracts were appropriately diluted and analysed by LC-MS/MS or FIA-MS/MS. Metabolites from LC-MS/MS part were quantified by isotopic dilution on a 7-point calibration curve, and metabolites from FIA-MS/MS were quantified by their relative intensities over the chosen isotopically labelled internal standards (semiquantitative approach). Finally, the intraplate correction was achieved by QC (quality control) normalisation using the QC2 level sample (analysed in three technical replicates). More details of the method and metabolites annotation have been published in our previous studies [27, 28].

The QC2 level sample (medium concentration level) was used to estimate the coefficient of variation (CV, expressed in %) as a marker of the reliability of measurement for each metabolite (metabolites with a CV > 25% were excluded). The limit of quantification (LOQ) and the limit of detection (LOD) for metabolites from LC-MS/MS analysis and FIA-MS/MS analysis, respectively, were other reliability criteria, and metabolites with more than 50% of values under the LOQ or LOD in each group were excluded.

Fold changes (FCs) of metabolite concentrations have been calculated as the ratio between two group means (HDFa vs. ADMSc, HDFa vs. DPSCs and DPSCs vs. ADMSc). Metabolites with FC > ± 1.5 were considered significantly changed. The q-values in the univariate analysis were calculated using the one-way ANOVA followed by Tukey’s HSD post-hoc analysis. Metabolites with q-values less than 0.05 were considered statistically significant. Metabolites selected by ANOVA followed by Tukey’s HSD post-hoc analysis were visualized as a heatmap. For multivariate analysis, principal component analysis (PCA) was constructed to get an initial overview of the data.

The MetaboAnalyst 6.0 web-based platform was used to perform statistical evaluation of metabolomic data using the abovementioned methods [29].

### Statistical Analysis

Statistical analyses were performed using R (version 4.4.2) and the libraries in the References section. Response variables were summarized using the median, lower, and upper quartiles. Data distributions were visually inspected using histograms, QQ plots, boxplots, and spaghetti plots.

Linear mixed models with a random intercept for individual subjects were used for repeated measures data. For single measurements, linear models were applied. Model residuals were primarily inspected using quantile-quantile plots; quantile residuals from the DHARMa package were used for linear mixed models. Appropriate transformations of response variables were identified using the powerTransform() function from the car package.

Estimated marginal means were calculated using the emmeans package, and pairwise comparisons were performed with Tukey’s method for *p*-value adjustment. When response variables were transformed, estimated marginal means were back-transformed to the original scale [30–35].

## Results

### MSCs from All Sources Showed Similar Cell Morphology and Cell Cycle Dynamics

**Fig. 1:**
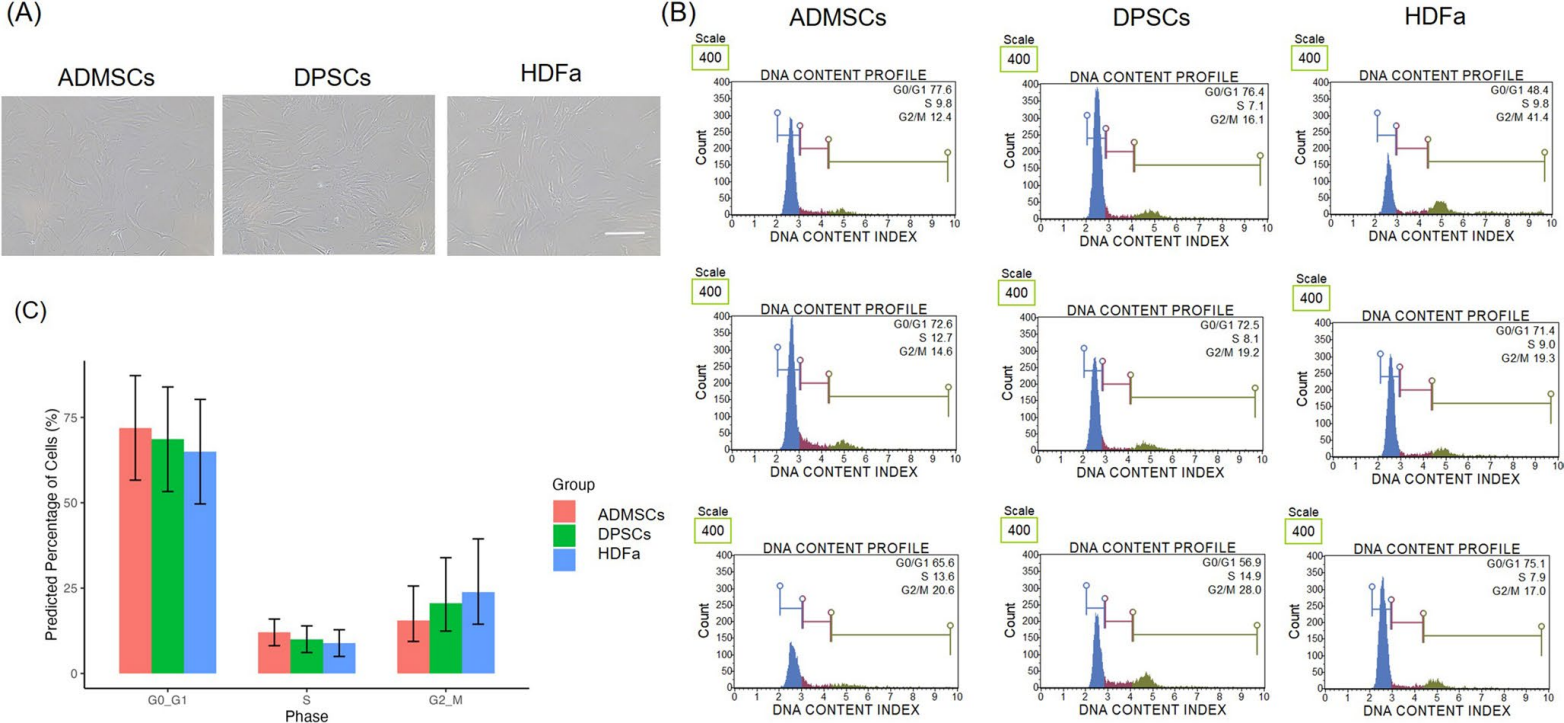
Cell morphology and cell cycle of adipose-derived mesenchymal stem cells (ADMSCs), dental pulp stem cells (DPSCs), and human dermal fibroblasts adult (HDFa). **A** Representative micrographs taken by phase contrast microscope at Magnification 100x, scale bar 100 μm. **B** Distribution in the cell cycle phases (G_0_/G_1_, S, G_2_/M) of HDFa, DPSCs and ADMSCs analyzed by the Muse Cell Analyzer using Muse Cell Cycle Assay Kit. Analyses of each donor. **C** The estimated Marginal means of cell cycle phases with 95% confidence intervals for each cell group

Before applying metabolomic analysis, we focused on confirmation the fundamental characteristics of mesenchymal stem cells according to ISCT. Cellular morphology is an essential parameter in cell characterization. The cells adhered to the plastic surface of the culture flasks. All three cell types, ADMSCs, HDFa, and DPSCs, displayed an elongated, spindle-shaped, and fibroblast-like morphology typical of MSCs with minor variations in elongation (Fig. 1). No significant changes in morphology were observed across the passages.

A microcapillary-based cell cycle analysis was performed to assess cell cycle dynamics under standard conditions. The distribution of cells across specific cell cycle phases, G0/G1, S, and G2/M, is presented in Fig. 1B and C with 65–72% of cells in the G0/G1 phase, 10% in S, and 16–26% in the G2/M phase. No significant differences were observed between the various cell types, and the data show some variability among donors. The calculated proliferation index was 34.9 ± 14.4% for HDFa cells, 31.1 ± 10.4% for DPSCs, and 28 ± 6% for ADMSCs.

### Cell Proliferation Analysis

**Fig. 2:**
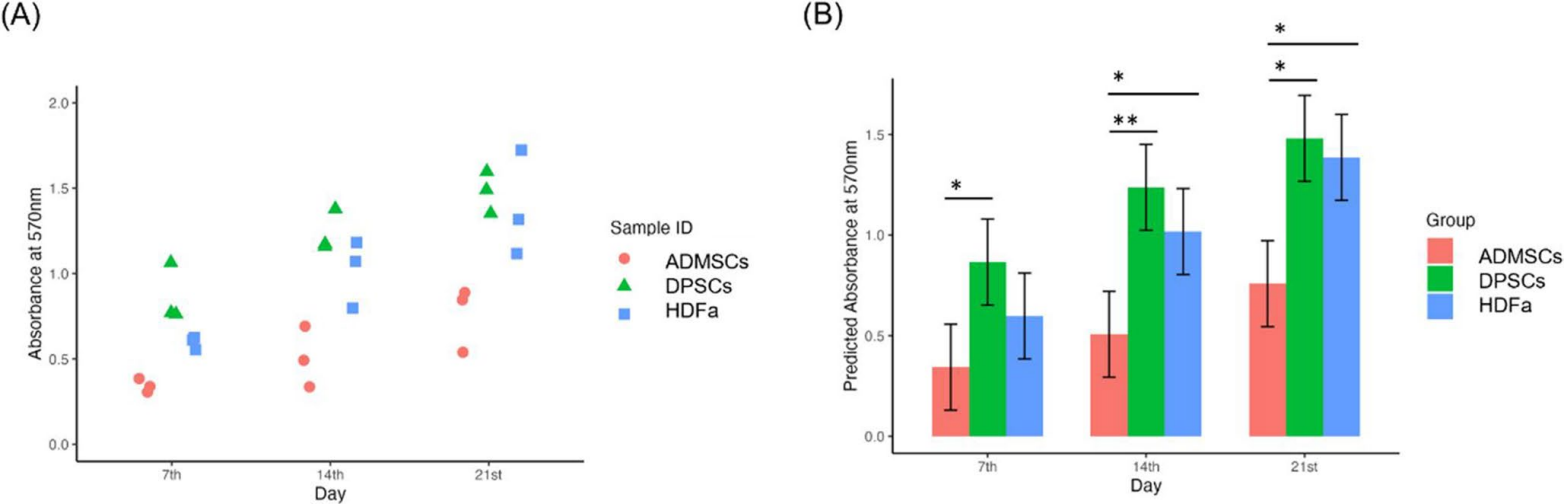
Proliferation of adipose-derived mesenchymal stem cells (ADMSCs), dental pulp stem cells (DPSCs), and human dermal fibroblasts (HDFa). **A** MTT assay on the 7th, 14th^,^ and 21 st day, the scatter plot showing individual absorbance measurements for each cell type in each donor. **B** The estimated Marginal means of absorbance with 95% confidence intervals for each cell group over time and (B). *p ˂ 0.05, ** *p* ˂ 0.005

Then, the MTT assay was performed to assess the metabolic activity of three cell types, HDFa, DPSCs, and ADMSCs at 7, 14, and 21 days post-culture. The MTT assay showed variability in the proliferation rates across cell types (Fig. 2). HDFa cells showed a consistent increase in absorbance over time, with the highest value measured on day 21. DPSCs also showed increased metabolic activity, reaching levels comparable to HDFa by day 21. In contrast, ADMSCs consistently exhibited lower absorbance values at all time points, indicating comparatively lower metabolic activity.

### Determination of Differentiation Potential

**Fig. 3:**
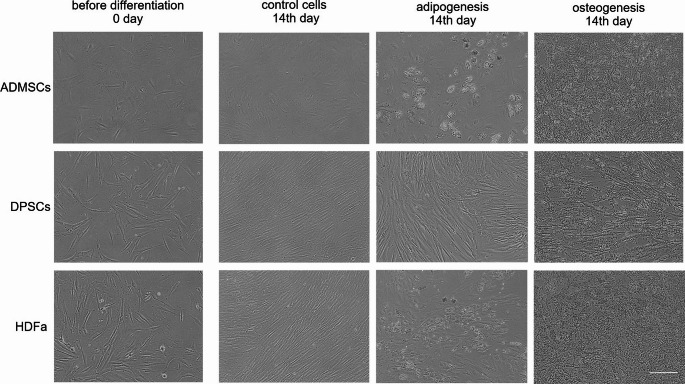
Representative micrographs of undifferentiated (control) and differentiated cells of adipose-derived mesenchymal stem cells (ADMSCs), dental pulp stem cells (DPSCs), and human dermal fibroblasts (HDFa) on the 14th day after adipogenic and osteogenic medium application. Magnification 100x, scale bar 100 μm

In parallel with the proliferation assay, the cells were cultured in differentiation media for 21 days to assess osteogenic and adipogenic differentiation ability. Morphological changes were observed throughout the differentiation, varying by both cell type and the differentiation lineage. When cells differentiated into adipocytes, intracellular lipid droplets formed, appearing as round vacuoles within the cells. When cells differentiated into osteoblasts, the mineralized extracellular matrix deposition was visible. Microscopic pictures confirmed successful differentiation (Fig. 3). The cells were stained on the 7th, 14th and 21 st day during the differentiation to compare their potential.

**Fig. 4:**
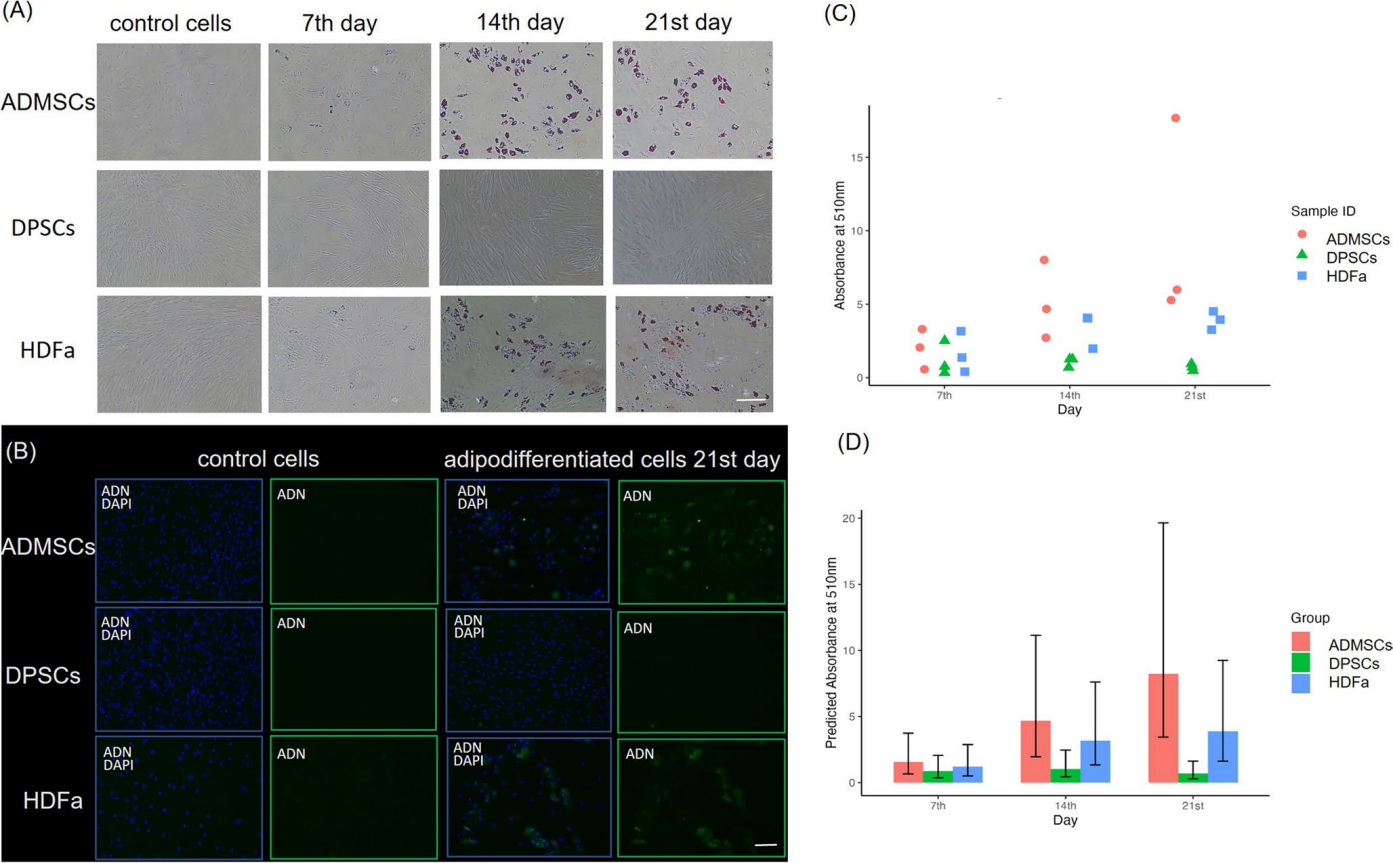
Oil Red O staining of lipid droplets accumulation in adipose-derived mesenchymal stem cells (ADMSCs), dental pulp stem cells (DPSCs), and human dermal fibroblasts (HDFa) at 7th, 14th, 21 st day during adipogenic differentiation. **A** Representative micrographs, Magnification 100x, scale bar 100 μm. **B** Immunocytochemical staining of undifferentiated and differentiated HDFa, DPSCs, and ADMSCs for presence of adipogenic marker: ADN – Adiponectin after 21 days post application of differentiation media. Expression of the proteins in the cytoplasm was detected by green fluorescence, and the nucleus was stained blue by DAPI. Representative micrographs. Magnification 100x, scale bar 100 μm. **C** Quantification of lipid droplets determined by Oil Red O staining, the scatter plot showing individual absorbance measurements for each cell type in each donor and (**D**) the estimated Marginal means of absorbance with 95% confidence intervals for each cell group over time. Absorbance data were normalized to the total amount of proteins isolated from fixed cells

Control cells showed a basic morphology for each cell type without any visible accumulation of Lipid droplets, as indicated by the low absorbance detected after Oil Red O application and subsequent destaining. Minimal Lipid droplet accumulation was observed in ADMSCs and HDFa cells at day 7. In contrast, DPSCs do not show any detectable Lipid droplets after staining, indicating that adipogenic differentiation was essentially not initiated. The consistent increased in absorbance after staining ADMSCs from day 7 to day 21 indicates a stable accumulation of Lipid droplets. On day 21, ADMSCs reached the highest level of adipodifferentiation, which confirmed their natural potential. A slight increase in absorbance after staining of HDFa was detected on days 14 and 21. However, the levels remain lower than those of ADMSCs, indicating a potential for adipogenic differentiation, but at a reduced level. Consistently low absorbance values were detected after staining HDFa with minimal variability, supporting the observation of limited lipid accumulation. The data points also show some sample variability, which may reflect biological variation in the potential for lipid droplet accumulation. Overall, ADMSCs show the highest adipodifferentiation over time, followed by HDFa, while DPSCs indicate low or negligible adipodifferentiation capacity under these conditions (Fig. 4).

The immunocytochemistry staining was performed using specific antibodies, such as adiponectin, FoxO1, and osteopontin, to confirm the adipogenic and osteogenic differentiation. After cultivation in differentiation media for 21 days, in HDFa and ADMSCs, we observed the presence of adiponectin, while in DPSC cells, no positive signal was detected (Fig. 4).

**Fig. 5:**
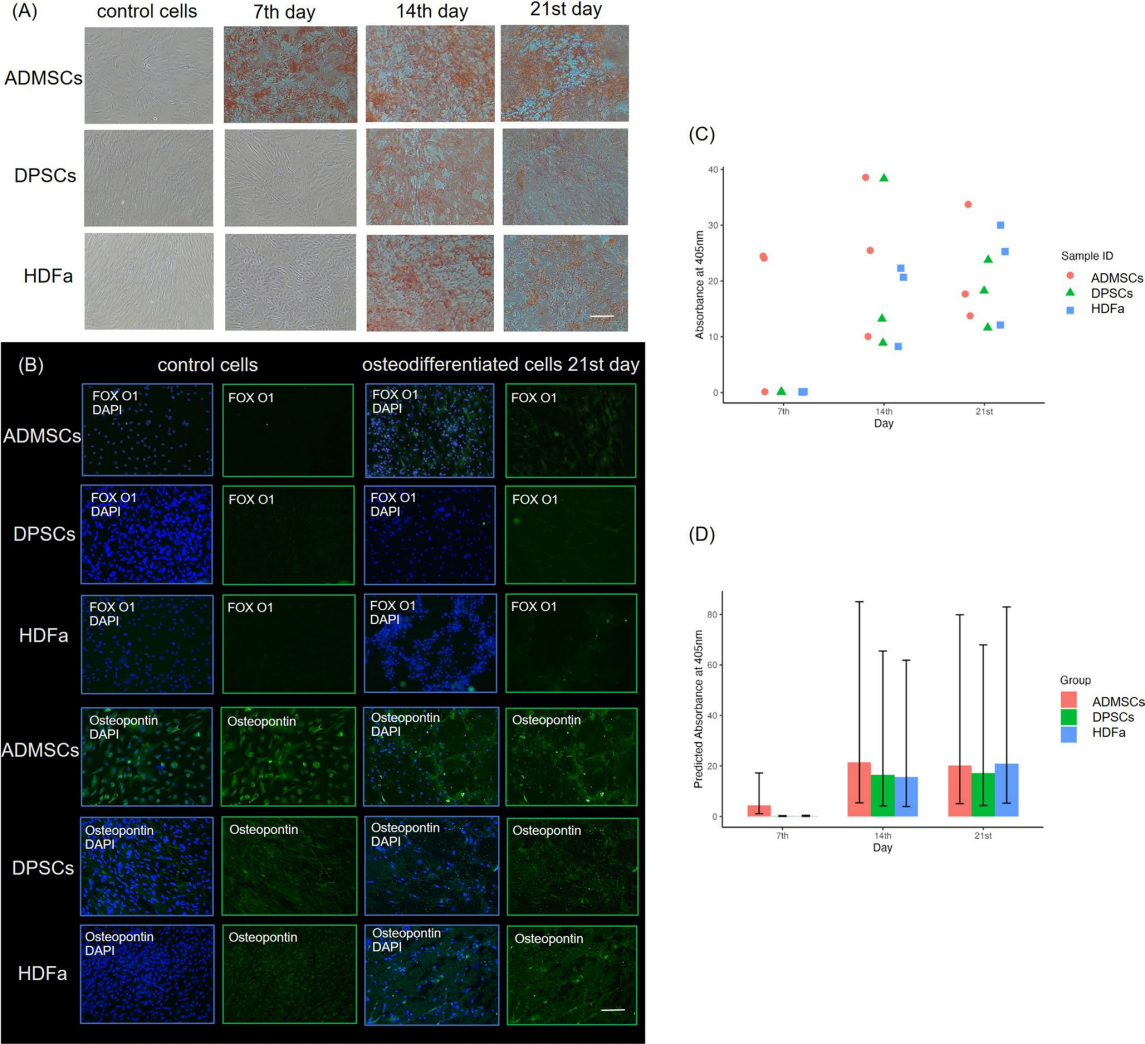
Alizarin Red staining of calcium deposits in adipose-derived mesenchymal stem cells (ADMSCs), dental pulp stem cells (DPSCs), and human dermal fibroblasts (HDFa) on the 7th, 14th, and 21 st day during osteogenic differentiation, **A** representative images, Magnification 100x, scale bar 100 μm. **B** Immunocytochemical staining of differentiated ADMSCs, DPSCs, and HDFa for the presence of osteogenic markers: FoxO1, Osteopontin after 21 days post application of differentiation media. Expression of the proteins in the cytoplasm was detected by green fluorescence, and the nucleus was stained blue by DAPI. Representative micrographs. Magnification 100x, scale bar 100 μm. **C** Quantification of calcium deposits determined by Alizarin Red staining, the scatter plot showing individual absorbance measurements for each cell type in each donor and (**D**) the estimated Marginal means of absorbance with 95% confidence intervals for each cell group over time. Absorbance data were normalized to the total amount of proteins isolated from fixed cells

Osteogenic differentiation was determined in each of the three types of cells. The quantification of calcium deposits stained with Alizarin Red corresponded with microscopic observations (Fig. 5). Images of the control cells displayed baseline morphology with no visible signs of differentiation and extracellular Matrix deposition. The data indicated that ADMSCs exhibit the highest differentiation or extracellular Matrix formation level. By the 7th day, ADMSCs showed initial signs of differentiation, while HDFa and DPSCs displayed only Limited morphological changes. By days 14 and 21, all cell types demonstrate progressively increased calcium deposit formation.

The expression of FoxO1 was detected in differentiated cells after 21 days post-induction. Determination of adiponectin and FoxO1 were absent in control cells. The osteopontin expression in differentiated and control cells was detected in all three cell types. While, in control cells, expression was observed within the cell, in differentiated cells, it was localized within newly formed calcificates. DAPI staining indicated the presence of cell nuclei (Fig. 5).

### Measurement of Mitochondrial Respiration Determined DPSCs as Cells with the Highest E-R Reserve

**Fig. 6:**
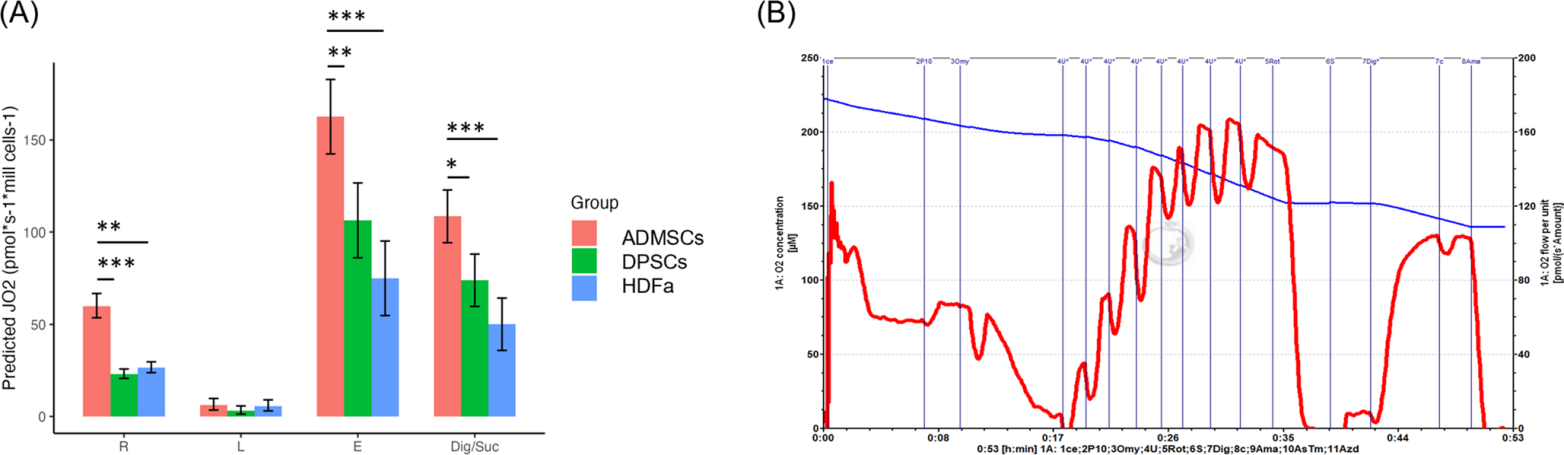
Respiratory states of adipose-derived mesenchymal stem cells (ADMSCs), dental pulp stem cells (DPSCs), and human dermal fibroblasts (HDFa). **A** Estimated Marginal means with 95% confidence intervals for each cell group. R - ROUTINE, L - LEAK, E - electron transfer capacity, Dig/Suc - Succinate driven respiration corrected for residual oxygen consumption, *p ˂ 0.05, **p ˂ 0.005, ***p ˂ 0.001. **B** Representative trace of respiration and O_2_ concentration in the protocol with living ADMSCs. The red line represents oxygen flux expressed in pmol/s per 10^6^ cells. The blue line represents the oxygen concentration in the oxygraph chamber in µmol/l. P – pyruvate, Omy – oligomycin, U – uncoupler, Rot – rotenone, S – Succinate, Dig – digitonin, c – cytochrome, Ama – Antimycin A

The mitochondrial respiratory functions were determined using high-resolution respirometry (HRR). Our data showed that the ROUTINE (R) respiration of ADMSCs was significantly higher than DPSCs and HDFa. After titration of oligomycin, all cell types exhibited low and similar LEAK (L) respiration. After adding CCCP, mitochondrial respiration was uncoupled, and ADMSCs showed significantly higher maximal electron transfer capacity (E) than DPSCs and HDFa. Differences were also observed at succinate-driven respiration (Fig. 6).

ATP-coupled respiration was calculated by subtracting the value of LEAK respiration from the value of ROUTINE respiration (R-L). Spare respiratory capacity was calculated by subtracting the ROUTINE respiration value from the maximal electron transfer capacity (E-R) (Table 1).

**Table 1:** Calculated respiratory characteristics of adipose-derived mesenchymal stem cells (ADMSCs), dental pulp stem cells (DPSCs), and human dermal fibroblasts (HDFa). Results are presented as mean ± sd

Cells	ATP-coupled respiration *R*-L	Spare respiratory capacity E-*R*
	(pmol*s^−1^*mill cells^−1^)	(pmol*s^−1^*mill cells^−1^)
ADMSCs	53.84 ± 7.50	102.54 ± 5.92
DPSCs	19.94 ± 1.20	83.32 ± 12.18
HDFa	20.68 ± 1.87	48.43 ± 16.58

The flux control ratios (FCR): ROUTINE flux control ratio R/E, netROUTINE flux control ratio (R-L)/E, and flux control efficiencies (FCE): E-L coupling efficiency 1-L/E, E-R reserve efficiency 1-R/E were calculated. The results are summarized in Table 2. Determination of flux control ratios offers the benefit of internal normalization, enabling the assessment of respiratory parameters independently of cell size and mitochondrial content. Since the ROUTINE flux control ratio is normalized to the Maximal capacity of the respiratory chain, it reflects the efficiency of mitochondrial respiration. The results demonstrated that approximately 30% of the ET capacity in HDFa and ADMSCs is Linked to ATP production, whereas in DPSCs, only 20% is utilized for this purpose. The efficiency of the E-R reserve is driven by intermediary metabolism and aerobic energy needs of the cells and is regulated by the efficiency of E-L coupling. DPSCs demonstrated the highest reserve efficiency capacity, followed by HDFa and ADMSCs. This indicated that DPSCs could cover increased energy demands above the basal level, thereby effectively responding to acute cellular stress or increased workload, thereby reducing the risk of ATP crisis.

**Table 2:** Flux control ratios and flux control efficiencies normalized for ET capacity as an internal normalization to express respiration independent of cell count. Results are presented as mean ± sd

Cells	ROUTINE flux control ratio	netROUTINE flux control ratio	E-L coupling efficiency	E-*R* reserve efficiency
	*R*/E	(*R*-L)/E	1-L/E	1-*R*/E
ADMSCs	0.37 ± 0.02	0.33 ± 0.03	0.96 ± 0.01	0.63 ± 0.07
DPSCs	0.22 ± 0.02	0.19 ± 0.02	0.97 ± 0.01	0.88 ± 0.02
HDFa	0.37 ± 0.07	0.29 ± 0.10	0.97 ± 0.01	0.63 ± 0.07

### Metabolomic Analysis

**Fig. 7:**
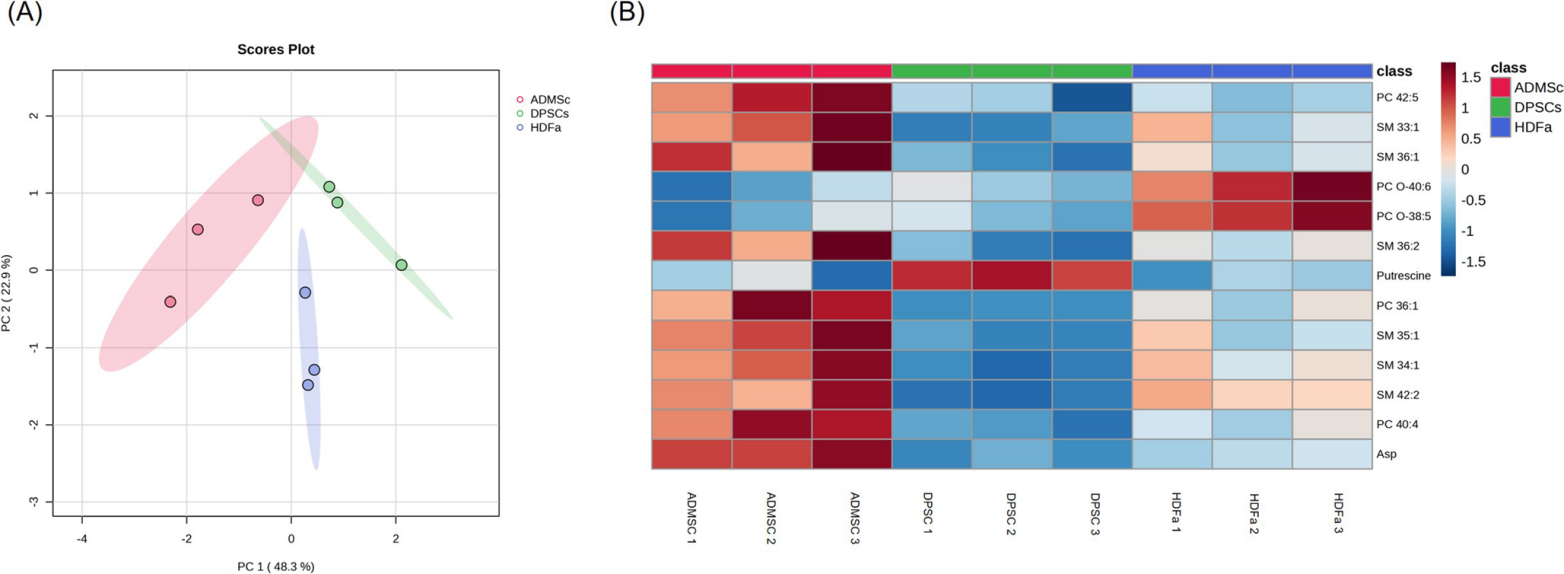
Metabolomic analysis of adipose-derived mesenchymal stem cells (ADMSCs), dental pulp stem cells (DPSCs), and human dermal fibroblasts (HDFa). **A** PCA plot of analysis of cell lysates. **B** Normalized concentrations of statistically significant metabolites in cell lysates presented in the form of a heat map (columns represented the cell types, and rows represented the metabolites) or in the form of box plots (**C**)

Finally, metabolomic analysis has been done to investigate the metabolic profiles of both cell lysates and media. Metabolites were identified and analyzed using AbsoluteIDQ^®^p180 kit (Biocrates Life Science) for targeted metabolomic analysis. Principal component analysis (PCA) was applied to the metabolite dataset obtained from lysate analysis (Fig. 7A). The data showed a clear separation of cells from their origins in cell lysates based on measured metabolites.

**Fig. 8:**
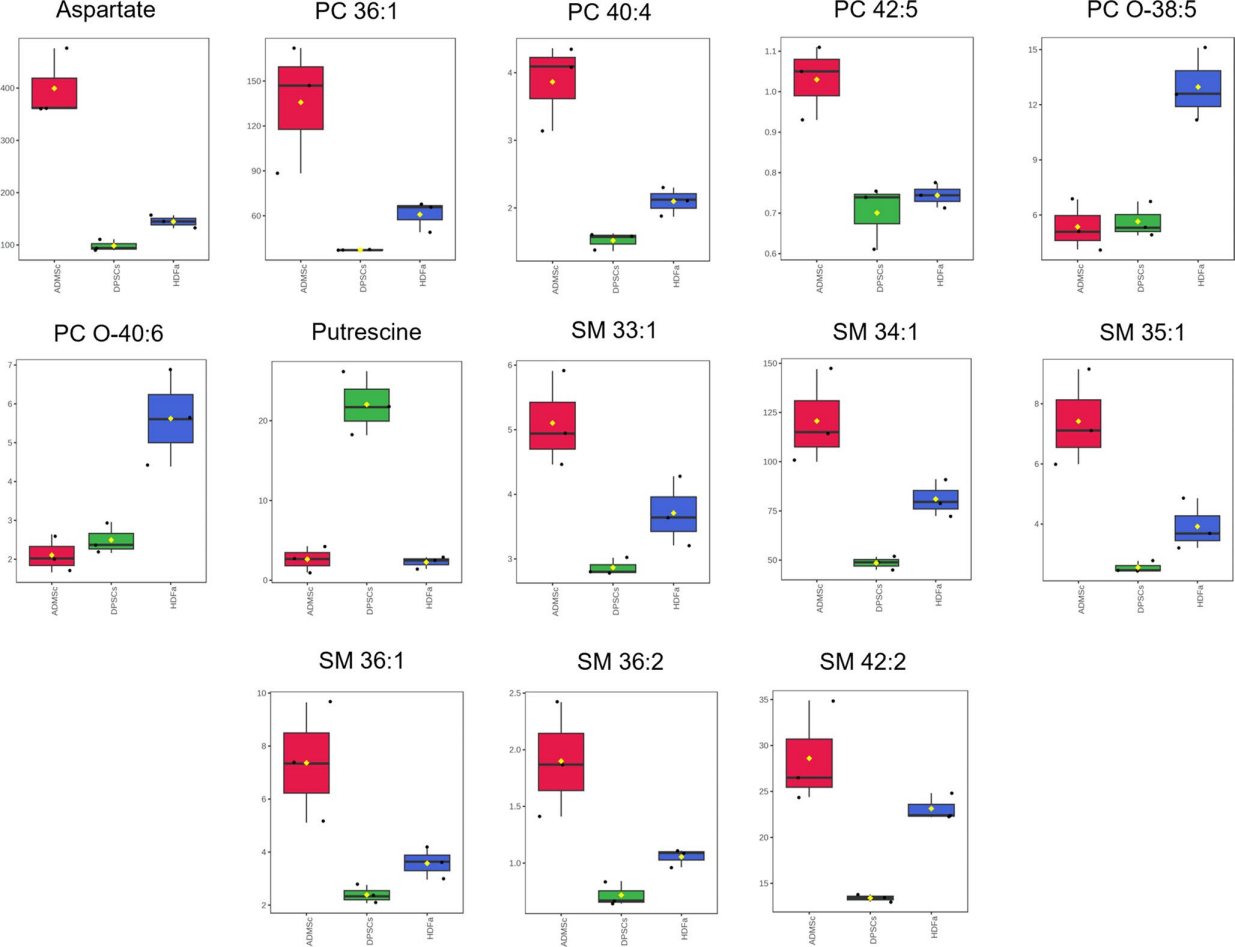
Boxplots of metabolite concentrations (µM) from Table 3. Boxplots exhibit the medians of the data (thick horizontal line), the means of the data (yellow diamond), the upper and lower hinges, a version of the upper and lower quartile (upper and lower line segment in the box), and the whiskers from minimum to maximum. Each boxplot is accompanied by a swarm plot of the data

Statistically significant metabolites were selected based on followed parameters: ANOVA (FDR corrected *p*-value ˂ 0.05), followed by Tukey’s post hoc test (*p* ˂ 0.05). Results are presented in Table 3, and normalized concentrations of metabolites are shown in the form of a heat map (Fig. 7B). Cell lysates demonstrated 13 significant metabolites specific to a particular cell type. Lysates of DPSCs showed a different profile with dedicated metabolites, Like putrescine, being highly expressed, distinguishing them from ADMSC and HDFa lysates. Also, sphingomyelin SM 34:1 was downregulated in DPSCs compared to HDFa and ADMSCs, respectively (−1.67-fold and − 2.48-fold decreased, respectively). Significant differences were observed in the aspartate level, up-regulated in ADMSCs (2.76-fold increase and 4.06-fold increase compared to HDFa and DPSCs, respectively) (Table 3; Fig. 8). Phosphatidylcholines (specifically PC O-38:5 and PC O-40:6) are upregulated in HDFa, which makes them characteristic of fibroblasts. Phosphatidylcholines (specifically PC 40:4, PC 36:1) and sphingomyelins (specifically SM 36:1 SM 35:1) were observed as upregulated in ADMSCs when compared to other cell types.

**Table 3:** Statistically significant metabolites (bold highlighted) determined in lysates of adipose-derived mesenchymal stem cells (ADMSCs), dental pulp stem cells (DPSCs), and human dermal fibroblasts (HDFa)

Metabolite	Anova *p*-value	Anova q-value (FDR-corrected)	Tukey’s HSD significant pairs (*p* ˂0.05)	Fold change		
				HDFa/ADMSc	HDFa/DPSCs	DPSCs/ADMSc
Aspartate	2.24E-05	3.76E-03	HDFa-ADMSc; DPSCs-ADMSc; DPSCs-HDFa	−2.76	1.47	−4.06
PC 40:4	3.38E-04	2.51E-02	HDFa-ADMSc; DPSCs- ADMSc; DPSCs-HDFa	−1.84	1.38	−2.54
SM 42:2	5.45E-04	2.51E-02	DPSCs-ADMSc; DPSCs-HDFa	−1.24	1.73	−2.13
SM 34:1	5.98E-04	2.51E-02	HDFa-ADMSc; DPSCs-ADMSc; DPSCs-HDFa	−1.49	1.67	−2.48
SM 35:1	1.06E-03	3.56E-02	HDFa-ADMSc; DPSCs-ADMSc	−1.89	1.53	−2.89
PC 36:1	1.43E-03	4.00E-02	HDFa-ADMSc; DPSCs-ADMSc	−2.23	1.64	−3.65
Putrescine	1.93E-03	4.14E-02	DPSCs-ADMSc; DPSCs-HDFa	−1.16	−9.74	8.36
SM 36:2	2.00E-03	4.14E-02	HDFa-ADMSc; DPSCs-ADMSc	−1.80	1.47	−2.65
PC O-38:5	2.33E-03	4.14E-02	HDFa-ADMSc; DPSCs-HDFa	2.42	2.30	−1.05
PC O-40:6	2.58E-03	4.14E-02	HDFa-ADMSc; DPSCs-HDFa	2.67	2.25	1.19
SM 36:1	2.71E-03	4.14E-02	HDFa-ADMSc; DPSCs-ADMSc	−2.06	1.50	−3.09
SM 33:1	3.36E-03	4.70E-02	HDFa-ADMSc; DPSCs-ADMSc	−1.38	1.29	−1.78
PC 42:5	3.74E-03	4.83E-02	HDFa-ADMSc; DPSCs-ADMSc	−1.38	1.06	−1.47

Abbreviations: *PC* Phosphatidylcholine (diacyl); *PC O* Phosphatidylcholine (acyl-alkyl); *SM* Sphingomyelin. More details of metabolite annotation have been published in previous studies [27, 28]

ANOVA, FDR corrected *p*.value ˂ 0.05, followed by tukey’s post hoc test (*p* ˂ 0.05) and │FC│≥ 1.5

We also analyzed the media’s metabolomic profiles, but no significant differences were detected. The media showed a more homogeneous profile than the lysates, suggesting a similar distribution of metabolites.

## Discussion

 In our study, we compared the functional properties of cells derived from three different origins: ADMSCs, DPSCs, and HDFa. We analyzed their differentiation potential, proliferation rates, cell cycle, respiratory activity, and metabolomic profiles. Understanding their behaviour and identifying similarities and differences could be key to optimize their use in cell-based therapeutic applications.

 Adipose-derived mesenchymal stem cells (ADMSCs) were successfully isolated from three donors. Other research teams have also observed similar morphological characteristics of ADMSCs [36–38]. All three cell types demonstrated strong adherence to culture flasks, retaining consistent morphology through passaging up to passage 5. They were characterized by an elongated and spindle-shaped morphology typical of MSCs, with slight differences in their degree of elongation [39–41]. MSCs have significant potential in clinical applications. However, during the long-term culture, they could lose their ability to differentiate multi-directionally and renew themselves. Binato et al. [42] reported that MSC cultures could be used successfully in cell therapy up to passages 4–5. They observed changes at the molecular level from passage 5 on, and they also admit that higher passage cells should be considered on a case-by-case. Passages 3–5 are used in several basic research studies.

Our results revealed differences in the proliferation rates among the cell types. HDFa showed a consistent increase in absorbance, reaching the highest levels on day 21. DPSCs also demonstrated a rise in metabolic activity, achieving values similar to HDFa by day 21. In contrast, ADMSCs displayed lower absorbance at all time points, indicating reduced metabolic activity compared to the other cell types. The previous studies comparing ADMSCs with different types of stem cells support this observation [43–45]. During the first six days, the metabolic activity of cells from different origins showed no significant differences (data not shown). The cell cycle was determined when cells reached approximately 80% confluence. Most cells (65–72%) were reported to reside in the G0/G1 phase, with 10% in the S phase and 16–20% in the G2/M phase, indicating that for cells at passage 4–5 an intermediate state of proliferation [46]. Bao et al. showed similar results (G0/G1 phase) in a study they where compared earlier (passages 3–4) and later (12–13) passages of human bone marrow-derived mesenchymal stem cells (hBMMSCs) expanded in vitro [47]. While no significant differences were observed among the cell types, some variability was noted between donors.

Both types of differentiation were performed in parallel. It is important to monitor adipogenesis and osteogenesis simultaneously because adipogenic factors suppress osteogenesis, while osteogenic factors, in turn, inhibit adipogenesis [48]. The cells showed adipogenic and osteogenic differentiation properties. Quantification of stained calcium deposits or Lipid droplets was consistent with microscopic observations. ADMSCs displayed the highest Lipid accumulation, followed by HDFa, with DPSCs showing no or minimal adipogenic differentiation under reported conditions. Our results confirm, previous findings that DPSCs do not differentiated into adipocytes after 21 days [49–51]. Some sample variability was noted, likely reflecting biological differences in lipid accumulation potential. Again, ADMSC cells exhibited the highest extracellular Matrix accumulation on the 7th day. Similar results were observed by Jin et al. [43]. On days 14 and 21, all cell types exhibited progressively higher calcium deposit formation, indicating increasing levels of osteogenic differentiation over time, which is also supported by other studies [10, 43, 52, 53]. The data more commonly indicated differences between individual donors rather than cell types. All data we obtained from staining were normalized to the total amount of proteins.

Conclusions based only on unnormalized data could be subjective. The method implemented in this way can produce artefacts if the dye needs to be adequately washed or filtered before staining. We detected proteins specific to both processes by immunocytochemical staining to confirm adipogenic and osteogenic differentiation. Based on the results after 21 days of differentiation, HDFa and ADMSCs showed positive determination of adiponectin in the extracellular matrix surrounding the lipid droplets as an indicator of successful adipogenic differentiation, while no signal was detected in DPSCs. FoxO1 expression was observed in osteodifferentiated cells after 21 days of induction. In a previous study, Nováková et al. identified members of the FoxO1 signaling pathway in osteodifferentiated cells using the label-free LC-MS method [54]. As described by Ma et al., FoxOs improve bone formation by increasing osteoblast differentiation at the expense of adipocyte differentiation [55]. In MSCs, the bidirectional effect of FoxOs on balancing osteogenic differentiation was discovered [56]. While nuclear staining indicates active FoxO1, which is common during oxidative stress and the early stages of differentiation, cytoplasmic staining corresponds to inactivated FoxO1 [57, 58]. Osteopontin expression was detected in differentiated and control cells across all three cell types. In control cells, the expression was observed within the cytoplasm, whereas in differentiated cells, it was localized in the extracellular space and mineralized calcifications, indicating advanced differentiation. These findings are consistent with previous studies [59–61].

The present study also showed that ADMSCs exhibited significantly higher ROUTINE (R) respiration than DPSCs and HDFa. All cell types displayed low and comparable LEAK (L) respiration. ADMSCs demonstrate significantly higher maximal electron transfer capacity (E) than DPSCs and HDFa cells. Calculated ATP-coupled respiration was the highest level in ADMSCs. Comparable results for HDFa cells were recorded, consistent with findings reported by other research groups [62, 63]. The results, summarized in Table 2, showed that approximately 30% of the electron transfer (ET) capacity in HDFa cells and ADMSCs was Linked to ATP production, compared to only 20% in DPSCs. The E-R reserve efficiency, which reflects the cells´ ability to respond to increased energy demands, was highest in DPSCs, followed by HDFa and ADMSCs. These results indicate that DPSCs can meet elevated energy demands during cellular stress or increased workload, reducing the risk of ATP depletion. Reserve efficiency capacity can predict how well the cell might deal with acute cellular stress [64]. In interpreting our results, it is also important to consider the embryological origin of these cells. DPSCs, derived from the neural crest, possess unique metabolic adaptations such as higher respiratory reserve and reduced adipogenic potential compared to mesoderm-derived ADMSCs and HDFa. This may reflect their specialized roles and suggests potential for applications in neuroregeneration and dental tissue engineering [65].

Finally, this study compares the metabolomic profiles of cell lysates, revealing significant metabolic variability, with certain metabolites being specific to particular cell types. To the best of our current knowledge, only a few studies have compared the metabolomic profiles of stem cells with one another. Putrescine concentration was significantly increased in DPSCs lysates compared to ADMSCs (8.36–fold increase) and HDFa (9.74-fold increase). This metabolite belongs to polyamines. How these molecules affect numerous cellular functions remain unclear. Polyamine biosynthesis is upregulated in proliferating cells, while its inhibition leads to cell cycle arrest. During differentiation, endogenous polyamine level decrease [66]. It was previously demonstrated that the exogenous application of polyamines, including putrescine, regulates osteogenic and adipogenic gene expression and promotes osteodifferentiation in hBMMSCs [67, 68]. Interestingly, although in our study, DPSCs naturally showed higher putrescine endogenous concentration and higher respiratory reserve than HDFa and ADMSCs, their osteogenic differentiation was at the same level as the other two cell types. Otherwise, DPSCs showed higher proliferation than other cell types during 21 days. Upregulated aspartate was observed in ADMSc compared to different cell types. Phosphatidylcholines (e.g., PC O-38:5, PC O-40:6) were upregulated in HDFa lysates, distinguishing them from DPSCs and ADMSCs. On the contrary, phosphatidylcholines (e.g., PC 40:4 and PC 36:1) and sphingomyelins (e.g., SM 36:1, SM 35:1) were accumulated in ADMSCs lysates (Table 3). Burk et al. [69] reported phospholipid detection as a method suitable for profiling mesenchymal stem cells with the potential to distinguish them also from fibroblasts. They compared human and equine ADMSCs, human fibroblasts and human peripheral blood mononuclear cells. They detected candidate MSC markers, PE O-36:3 and PG 40:7, as potential markers across various culture conditions and PE O-36:3 across origins. Sphingomyelins (SMs) are the most abundant sphingolipids in the membrane of mammalian cells. DeVeaux et al. [70] demonstrated that MSCs derived from mature tissue sources such as ADMSCs may retain characteristic sphingolipid profiles and cellular properties. In conclusion, lipidomics represents a potential tool for MSC profiling. Pradas’ studies on rat tissues showed a distinct lipid “fingerprint” for each tissue [71]. Furthermore, while our study focused on functional and metabolic assessments, the immunomodulatory properties of these cells are relevant in regenerative medicine.

Metabolism also plays critical roles in the MSCs’ immunomodulatory properties. MSCs exert their immunomodulatory properties by secreting multifunctional molecules mainly via paracrine mechanisms. These molecules such as cytokines, growth factors, or extracellular vesicles. influence the behavior of immune cells, thereby regulating immune responses [72]. MSCs from different sources can vary in secretome composition, which could influence their therapeutic efficacy and safety in allogeneic transplantation settings [73–77]. Recent research has also demonstrated that MSCs’ immunomodulatory properties may be influenced by metabolism - manipulating metabolism could be a promising strategy to enhance their therapeutic effectiveness [78]. Campos et al. suggested that the immune and anti-inflammatory capacities of MSCs might be associated also with lipid metabolism - after pro-inflammatory stimulation, MSC lipidomic profile has been changed [79]. In tissues demanding high level of energy, PPARβ/δ increases the expression level of genes associated with fatty acid transport and β-oxidation. On the other hand, Lopez et al. showed that PPARβ/δ manipulation in MSCs exerted anti-inflammatory effects [80]. In conclusion, although the lipid metabolism probably plays a critical role in the immune regulation, some results remain controversial and further research is needed in this area.

## Conclusion

Our results demonstrated the unique characteristics and functional capabilities of ADMSCs, DPSCs, and HDFa in terms of morphology, metabolic activity, differentiation potential, and mitochondrial and metabolomic profiles. While all three cell types shared similar standard MSC features, they also exhibited distinct differences in proliferation, differentiation efficiency, mitochondrial reserve capacity, and metabolomic profiles, emphasizing their specific biological roles and potential cell-based applications. HDFa may represent a promising alternative to mesenchymal stem cells for use in cell therapies.

In conclusion:

1. ADMSCs displayed lower metabolic activity than DPSCs and HDFa. They showed the best adipogenic and osteogenic differentiation potential during the initial days after applying corresponding media. Aspartate, several phosphatidylcholines (PC 40:4, PC 36:1) and sphingomyelins (SM 36:1, SM 35:1) were upregulated in ADMSCs lysates, which made ADMSCs different from DPSCs and HDFa cells.
2. DPSCs showed the highest E-R reserve efficiency. The putrescine concentration was determined to be significantly higher in DPSCs lysates. These two characteristics distinguish them from ADMSCs and HDFa.
3. HDFa showed very similar characteristics to other MSCs across the studied parameters and may be a suitable alternative. Phosphatidylcholines (PC O-38:5, PC O-40:6) were upregulated in HDFa lysates, distinguishing HDFa from DPSCs and ADMSCs.

### Limitations of study

 The study was conducted on only three samples from each cell source. Studies on a more significant number of patients are needed to characterize the cells more thoroughly and to confirm possible distinguishing molecules

## Data Availability

All data analyzed during this study are included in this published article. The generated datasets are available from the corresponding author upon reasonable request.
